# A Multi-Parameter Inspection Platform for Transparent Packaging Containers: System Design for Stress, Dimensional, and Defect Detection

**DOI:** 10.3390/s25247531

**Published:** 2025-12-11

**Authors:** Huaxing Yu, Zhongqing Jia, Chen Guan, Zhaohui Yu, Xiaolong Ma, Xiangshuai Wang, Bing Zhao, Xiaofei Wang

**Affiliations:** Shandong Key Laboratory of Optoelectronic Sensing Technologies, National-Local Joint Engineering Laboratory for Energy and Environment Fiber Smart Sensing Technologies, Laser Institute, Qilu University of Technology (Shandong Academy of Sciences), 3501 Daxue Road, Changqing District, Jinan 250353, China; 10431230165@stu.qlu.edu.cn (H.Y.); jiazhongqing@vip.sdlaser.cn (Z.J.); guanchen1993@qlu.edu.cn (C.G.); 19854106335@163.com (Z.Y.); 15653990653@163.com (X.M.); w18865708405@163.com (X.W.); zhaobing@sdlaser.cn (B.Z.)

**Keywords:** residual stress, dimensional measurement, defect detection, machine learning, coordinated inspection

## Abstract

With increasing quality demands in pharmaceutical and cosmetic packaging, this work presents a unified inspection platform for transparent ampoules that synergistically integrates stress measurement, dimensional measurement, and surface defect detection. Key innovations include an integrated system architecture, a shared-resource task scheduling mechanism, and an optimized deployment strategy tailored for production-like conditions. Non-contact residual stress measurement is achieved using the photoelastic method, while telecentric imaging combined with subpixel contour extraction enables accurate dimensional assessment. A YOLOv8-based deep learning model efficiently identifies multiple surface defect types, enhancing detection performance without increasing hardware complexity. Experimental validation under laboratory conditions simulating production lines demonstrates a stress measurement error of ±3 nm, dimensional accuracy of ±0.2 mm, and defect detection mAP@0.5 of 90.3%. The platform meets industrial inspection requirements and shows strong scalability and engineering potential. Future work will focus on real-time operation and exploring stress–defect coupling for intelligent quality prediction.

## 1. Introduction

With the growing global attention to product quality and safety, transparent packaging containers (such as ampoules, reagent bottles, and plastic bottles) are being increasingly used in key industries including pharmaceuticals, food, and cosmetics. The specific demands of these industries require transparent containers not only to provide good sealing and safety but also to ensure product traceability and market acceptance. In the medical field, transparent packaging containers are essential components of drugs and medical devices, and their quality directly affects treatment outcomes and medication safety. The World Health Organization (WHO), in its Guidelines on Packaging for Pharmaceutical Products, clearly states that transparent containers help ensure the stability and safety of medicines while allowing healthcare professionals and patients to observe the condition of the drug [1]. Studies have shown that glass ampoules offer excellent chemical stability and interact minimally with drugs, making them widely used in pharmaceutical packaging [2]. In the food industry, transparent packaging has become an important trend in design due to its advantages in product display and information delivery, which help enhance consumer trust and sense of safety [3,4]. In the cosmetics industry, transparent containers are often made from chemically inert glass, which helps maintain the stability and effectiveness of the product while enhancing its visual quality [5]. These containers not only protect the formulation but also improve the product’s display appeal, making it more attractive and competitive in the market. In conclusion, transparent packaging containers play a vital role in the pharmaceutical, food, and cosmetics industries. Whether in protecting the safety of drugs and food or enhancing the market competitiveness of cosmetic products, the quality of transparent containers directly affects the overall performance of the product and consumer acceptance.

In modern industrial manufacturing of transparent packaging containers, achieving high-precision and high-efficiency quality inspection has become a key direction in the development of intelligent manufacturing. This is especially important in industries such as pharmaceuticals, food, and cosmetics, where packaging quality is critical. Transparent containers not only serve the basic function of protecting the contents, but their structural reliability and safety also directly affect product stability, market acceptance, and user safety. For example, transparent containers like ampoules, reagent bottles, and plastic bottles may experience breakage during use due to manufacturing defects, dimensional deviations, or abnormal residual stress, which can lead to quality issues such as drug leakage, contamination risks, or filling failures. Therefore, building a systematic and integrated inspection and analysis mechanism is essential for improving the overall quality and reliability of these containers. As highlighted in the Fall 2024 cover story of *Vision Spectra*: “Vision makes quick work of bottle inspection,” industrial vision is becoming a core enabling technology for bottle inspection [6].

It is important to note that stress detection, dimensional measurement, and defect recognition are not independent tasks in practical applications, but rather closely related physical processes. Traditional inspection procedures often rely on separate systems to perform each task individually, which leads to high costs, complex workflows, and fragmented information, ultimately limiting overall inspection efficiency and accuracy. Recent studies have shown significant interconnections and coupling among these three tasks. For example, reference [7] used finite element analysis to reveal that thin-walled glass containers like ampoules tend to develop stress concentrations in structural transition zones—areas that are not only potential starting points for microcrack propagation but may also affect local dimensional stability. Similarly, studies on polymer bottles [8,9] have shown that environmental stress cracking (ESC) often initiates from small defect areas and evolves along stress gradients, indicating a strong coupling between defects and internal stress. Dimensional measurement is also affected by defects: surface damage may alter the physical contour, interfering with the extraction of measurement boundaries; in addition, image noise and grayscale variation can degrade the stability and accuracy of edge detection algorithms. However, most existing studies focus on optimizing individual detection modules, while lacking system-level exploration of coordinated inspection of stress, dimension, and defects within a unified platform. Especially under resource-constrained conditions, achieving coordinated inspection with balanced accuracy, efficiency, and structural compactness remains a key challenge to be solved.

Therefore, future inspection systems should gradually shift from traditional functionally separated architectures to an integrated and multi-task coordinated inspection framework that combines defect, stress, and dimensional inspection. By fusing perception mechanisms with information processing chains, such systems can not only improve detection efficiency and stability, but also enable global modeling of container health conditions, thereby promoting the implementation of predictive maintenance and intelligent quality control.

In the field of stress detection, photoelasticity is one of the mainstream techniques, enabling visualization and quantitative analysis of residual stress based on the birefringence effect of materials. Errapart et al. achieved accurate measurement of residual stress distribution in non-axisymmetric glass containers using the principles of photoelasticity, demonstrating the feasibility and effectiveness of this method for inspecting glass products with complex shapes [10]. To enhance sensitivity, several emerging technologies have been developed in recent years, such as the scattered light polarimeter (SCALP) technique [11], quantum polarization imaging systems [12], and dual photoelastic modulator-based frequency-difference modulation methods [13]. However, these advanced methods often involve complex systems and high equipment costs, limiting their practical deployment in production lines. Efferz et al. [14], in their study on edge stress measurement of architectural tempered glass, also pointed out that current systems still face adaptability bottlenecks in industrial environments, indicating the need for further development of efficient and low-cost inspection technologies. Against this background, the Senarmont compensation method [15], known for its simple structure, ease of operation, and low cost, remains widely used for stress detection in transparent containers. It is especially suitable for frequent sampling inspections under resource-constrained conditions. Therefore, we selected the Senarmont compensation method as the most appropriate approach for stress measurement in this work.

In the area of dimensional measurement, machine vision offers a non-contact and automated solution with high precision. Li [16] developed a system for measuring the dimensions of shaft parts based on an improved single-pixel edge detection method. Miao [17] achieved online dimensional measurement of disk-shaped parts through camera calibration and geometric fitting. Zhou and Hartman [18] proposed a cost-effective vision inspection system for measuring key dimensions of plastic bottles, using intelligent image processing techniques to achieve high measurement accuracy. Eshkevari et al. [19] designed a glass bottle inspection approach for pharmaceutical use, which improved the accuracy of complex boundary detection through image segmentation. These studies demonstrate that machine vision has great potential in the dimensional inspection of transparent containers, especially in achieving high precision and automation.

In the field of defect detection, traditional methods such as rule-based template matching, edge detection, and statistical thresholding were widely used in early applications. For example, Zhou et al. [20] proposed an automated glass bottle bottom inspection system based on machine vision, using saliency detection and template matching to identify bottom defects. Yang et al. [21] applied Halcon software with threshold segmentation and edge detection techniques to achieve high-precision, high-speed, and stable detection of ampoule bottle mouth defects. However, due to the high reflectivity, complex lighting conditions, and curved surfaces of transparent materials, these rule-based methods face challenges and have gradually shown limitations such as poor robustness and weak generalization. To overcome these issues, deep learning methods have rapidly emerged in industrial vision. Models based on convolutional neural networks (CNN) can automatically extract image features, significantly improving the ability to recognize diverse types of defects.For instance, Kazmi [22] proposed a deep CNN-based framework for visual inspection of plastic bottles, demonstrating high accuracy and low resource consumption. Claypo [23] combined CNN-LSTM with instance-based classification to further improve the accuracy and efficiency of surface defect detection in glass bottles. These studies highlight the strong potential of deep learning in complex industrial environments. It is worth noting that in real industrial production, defect detection systems must not only achieve high accuracy but also meet requirements for real-time performance and high throughput. To address this, the YOLO (You Only Look Once) [24] family of algorithms, as a typical single-stage object detection approach, has gained significant attention due to its end-to-end structure, high parallelism, and millisecond-level detection speed. YOLO stands out for its ability to balance detection accuracy and real-time performance, making it an increasingly popular solution in industrial defect detection scenarios.

Although significant progress has been made in the individual detection of residual stress, structural dimensions, and surface defects, most industrial inspection systems still adopt a separated design, where the three types of tasks are handled by different modules or standalone devices. This architecture not only increases deployment costs and maintenance complexity, but also leads to fragmented data flows and limited information sharing, making it difficult to meet the actual demands for multi-parameter coordinated inspection and high-throughput sampling on production lines. Especially in resource-constrained environments, achieving compact structure, functional integration, and efficient multi-target detection remains a major challenge.

On the other hand, most existing studies focus on algorithm optimization for individual tasks, with limited attention to the design of mechanisms that enable coordinated execution of multiple inspection tasks within a unified system, as well as the unified scheduling of software and hardware resources. The lack of system-level integration and coupling capabilities has become a key bottleneck restricting the development of industrial vision systems toward high reliability and real-time performance.

To address the above challenges, this paper proposes a multifunctional vision inspection platform for transparent containers, using pharmaceutical ampoules as a representative example. For the first time, the platform integrates residual stress detection, key dimensional measurement, and surface defect recognition within a unified system architecture. The platform improves system integration and task coordination by designing a visual resource sharing mechanism and a module-level scheduling process, enabling dimensional and defect inspection to share a common vision subsystem while maintaining high-precision stress analysis capabilities.In the defect recognition module, the YOLOv8 deep learning model is introduced, combined with optimized image acquisition and processing scheduling, to enable efficient detection of various defect types without increasing hardware load. This ensures the system’s overall real-time performance and adaptability to industrial environments. The integrated architecture not only enhances multi-parameter inspection efficiency but also provides a data foundation for modeling the relationships among defects, stress, and structural features, showing strong potential for industrial application and future research development.

To address the fragmented nature of existing inspection systems, we propose a unified vision-based platform and introduce innovations across three technical dimensions:

(1) System Architecture:We propose a unified visual inspection platform for ampoule quality control, integrating stress evaluation, dimensional measurement, and defect detection into a single imaging system. Dimensional measurement and defect detection share the same planar backlight and camera architecture, enabling synergistic multi-parameter inspection. To the best of our knowledge, this is the first multi-functional integrated platform applied to ampoule quality control, filling the gap in integrated ampoule inspection.

(2) Task Scheduling Mechanism: We design an industrial real-time multi-task scheduling strategy for shared imaging modules, coordinating dimensional measurement and defect detection via dynamic regulation of acquisition timing, exposure switching, and processing sequences. This strategy achieves efficient resource utilization and real-time responsiveness, addressing the conflict and latency issues in multi-task industrial inspection.

(3) Deployment Strategy:We develop a module-level deployment strategy with hardware-software co-optimization to address key challenges of inference latency, illumination stability, and industrial environment adaptability. This strategy ensures robust and efficient system operation under near-production conditions, enhancing the practical applicability of the proposed platform.

In addition, we built a simulated production-line testing setup to evaluate the overall performance of the integrated platform. The evaluation shows that the system simplifies hardware requirements while preserving the precision needed for stress, dimensional, and defect inspection. In our tests, the platform achieved an optical path difference error of about ±3 nm for stress measurement, a dimensional accuracy of ±0.2 mm, and an mAP@0.5 of 90.3% for defect detection, indicating its suitability for industrial deployment and scalable integration.

## 2. Experimental System

**Figure 1:**
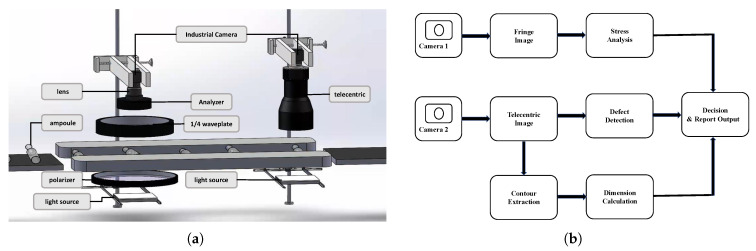
System architecture and data-processing pipeline of the proposed inspection platform. (**a**) overall hardware layout with functional modules; (**b**) Block diagram of the data-processing pipeline with two camera paths and task flow.

To achieve automated and high-throughput quality inspection of pharmaceutical ampoules, in this work, the design and implementation of a dual-module vision inspection platform that integrates stress analysis, dimensional measurement, and surface defect detection.The entire inspection workflow is presented in Figure 1, emphasizing the image acquisition, lighting control, task scheduling, and inference procedures across three parallel inspection modules.These two units share the same conveying mechanism and operate in a coordinated manner under a unified scheduling framework, enabling continuous multi-parameter inspection under online conditions.

The stress inspection module relies on a photoelastic optical setup to measure residual stress without contacting the ampoule. A vertical polarized-light configuration is used to capture fringe patterns, which are later interpreted by the stress-analysis algorithm.

Further along the line, dimensional measurement and defect inspection are carried out using a shared telecentric imaging system with backlighting. The telecentric design minimizes parallax and provides reliable contour and surface information for both tasks.

All modules are managed by the central control system, which handles conveyor motion, image triggering, and task scheduling. The stress and dimension/defect inspections run independently, and the latter reuses the same optics to avoid additional hardware. The final inspection results are integrated and forwarded to the MES for traceability and quality management.

## 3. Stress Detection

### 3.1. Measurement Principle

The photoelastic method is a non-contact optical technique for stress analysis, based on the birefringence exhibited by transparent materials under mechanical stress. When a transparent material such as glass is subjected to stress, its internal refractive index varies with the stress direction, resulting in optical anisotropy [25].

When linearly polarized light passes through a stressed material, it splits into two polarized beams vibrating in perpendicular directions. These two beams have different refractive indices and therefore travel at different speeds, causing a phase difference that leads to an optical path difference [26]. As the light passes through the subsequent polarized optical system, interference fringes are formed. The pattern of these fringes reflects the stress distribution in the material, with the brightness of the fringes directly related to the magnitude of the stress.

For glass ampoules, the relationship between the optical path difference, material stress, and geometric properties can be expressed by the following equation:

(1) Δ=C·σ·h

where Δ is the optical path difference, *C* is the photoelastic constant, σ is the stress, and *h* is the material thickness. By analyzing the change of the optical path difference as the analyzer rotates, the stress distribution characteristics of the ampoule can be derived.

### 3.2. Method

In this study, the Senarmont compensation method [15] was employed for stress measurement of ampoules due to its simple structure and high measurement sensitivity. The Senarmont method is widely used in commercial instruments for detecting residual stress in optical glass. In comparison, the Tardy compensation method [27] introduces a second quarter-wave plate based on the Senarmont principle, enabling adjustment of stress direction through isoclinic lines. This improves accuracy for specimens with uncertain or complex stress orientations but increases system complexity and operational difficulty. The Babinet compensator method [28] adjusts optical path difference by sliding a wedge-shaped crystal, offering high precision and suitability for laboratory studies; however, its high cost makes it less appropriate for large-scale applications.

**Figure 2:**
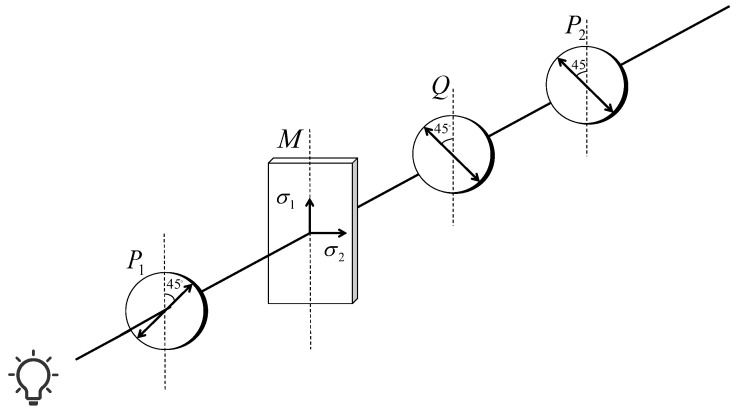
Senarmont-based quantitative stress measurement using polarimetric fringe analysis.

The stress measurement system based on the Senarmont compensation method mainly consists of a polarizer, a quarter-wave plate (λ/4 plate), and an analyzer, as shown in Figure 2. The polarization axes of the polarizer and analyzer are oriented at ±45° relative to the horizontal direction and are mutually perpendicular. The quarter-wave plate is placed between the sample and the analyzer, with its fast axis aligned parallel to the polarization direction of the polarizer and its slow axis parallel to that of the analyzer.

During the inspection process, the natural light emitted from the source is converted into linearly polarized light with a specific vibration direction after passing through the polarizer. The polarized light then successively passes through the glass sample under test and the quarter-wave plate (λ/4 plate). It should be noted that during the inspection process, the polarized light passes through the ampoule glass twice. The light first enters through the front wall of the glass, propagates through the interior of the ampoule, and then passes through the opposite wall before exiting. Due to the internal stress within the glass, the light undergoes a phase difference during both passes. As a result, the measured optical path difference corresponds to the combined effect of the double-pass, so that the accumulated optical path delay can be used to obtain the correct stress values. Due to the internal stress within the glass, the incident light is split into two orthogonal polarization components with a phase difference φ. After passing through the quarter-wave plate, this phase difference is further modified, forming elliptically polarized light with a certain ellipticity. When the light passes through the analyzer, the two components are recombined into linearly polarized light with the same vibration direction, producing interference fringes along the analyzer’s polarization axis. By rotating the analyzer, the variation in fringe intensity can be observed, and the optical path difference can be calculated from the rotation angle θ, which is then used to determine the stress within the sample [29].

The conceptual experimental diagram in Figure 2 uses a planar sample, and it serves only to illustrate the basic principle of the Senarmont compensation method. In actual measurements, ampoules are used as test samples. Their curved geometry can affect the propagation path of light within the samples to some extent. To reduce the interference of this geometric effect as much as possible, the ampoules are placed horizontally so that the light beam passes nearly perpendicularly through the bottle wall. The sidewall area of the ampoules is chosen as the measurement region, which ensures that the measured optical path differences can accurately reflect the local stress. Though the curved surface may cause small differences in the distribution of stress fringes compared with planar samples, the measurement results remain highly reliable through the above arrangement and calibration.

#### 3.2.1. Automatic Zero Adjustment Mechanism

To improve the accuracy of stress measurements and eliminate system errors, an automatic zero calibration is performed before each inspection. This procedure compensates for small assembly deviations in the light source, polarizer, quarter-wave plate, and analyzer, ensuring precise measurements. The goal of zero calibration is to achieve the minimum system output light intensity in the absence of a sample, thereby establishing the ideal configuration of the optical components.

**Figure 3:**
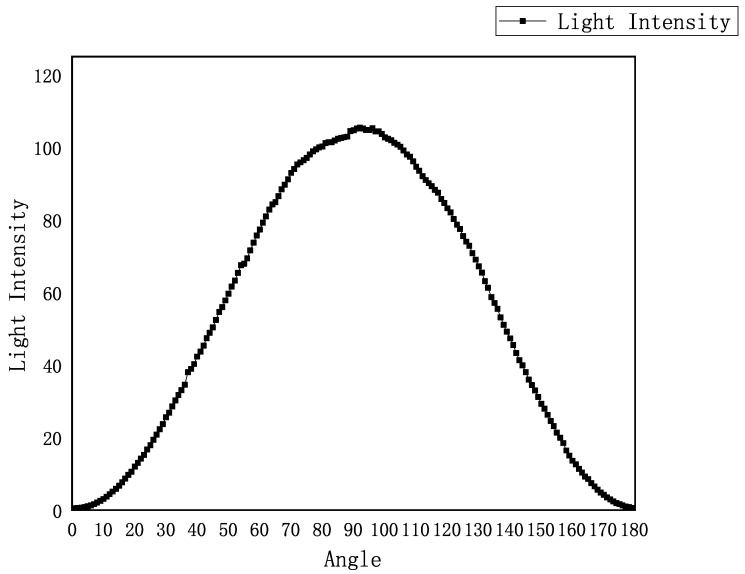
Intensity–angle response curve of polarized light under stress-induced birefringence.

During calibration, the ampoule is temporarily removed so that light propagation is influenced only by the internal optical elements. The system rotates the analyzer using a stepper motor and captures images at different angles, calculating the corresponding light intensity. From these measurements, a light intensity–angle response curve is generated, and the analyzer angle corresponding to the minimum light intensity is identified as the zero calibration position (Figure 3).

This automatic zero calibration mechanism effectively avoids human operation errors, improving the system’s repeatability and stability, and provides accurate and reliable initial conditions for subsequent stress measurements.

#### 3.2.2. Stress Calculation

1. Deflection Angle Acquisition via Computer Vision Methods

After system calibration, the optical setup operates in a dark-field imaging mode. When the ampoule sample is placed into the stress detection system, the initial image shows only a faint outline of the bottle without visible interference fringes. As the analyzer gradually rotates, the brightness of the bottle region changes, local interference fringes begin to appear, and the overall contour becomes clearer. During image processing, a sequence of images of the same bottle region (ROI) captured at different analyzer angles is analyzed. An edge detection method is applied to extract the local gray-level variation amplitude along the fringe boundaries, referred to as the edge amplitude. Since clearer fringes exhibit sharper gray-level transitions, the difference between the maximum and minimum edge amplitudes in each image is defined as the contrast value, serving as an indicator of fringe clarity. This metric reflects the range of gray-level variation between the strongest and weakest edges, effectively characterizing the change in fringe visibility. Based on this contrast index, the image with the largest edge amplitude difference is selected, and its corresponding analyzer angle is taken as the optimal stress analysis angle, providing the basis for subsequent optical path difference calculation.

**Figure 4:**
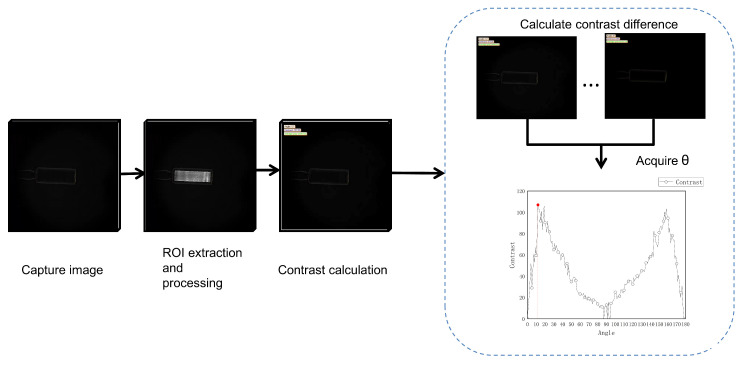
Flowchart of polarizer angle estimation. Each subfigure is labeled in the top-right corner with three key parameters: the rotation angle of the analyzer, the contrast value of the current image, and the average gray value of the current image.

Figure 4 shows the process of determining the optimal rotation angle of the analyzer in the stress detection system using a computer vision method. As the polarized light passes through the ampoule, the interference intensity changes periodically with the rotation of the analyzer. After every 180° rotation, the optical path difference and the bright–dark fringe pattern repeat once. To record a complete cycle of intensity variation, the analyzer is driven by a motor to rotate clockwise from 0° to 180°. This setup ensures that the collected stress images fully cover one polarization cycle, providing sufficient information for subsequent contrast analysis and optical path difference calculation.

During the rotation of the analyzer, the system captures a stress image every 1° and transfers it to the computer for processing. For each image, the bottle’s fringe distribution area is first extracted as the region of interest (ROI). The contrast of the image is then calculated as the difference between the maximum and minimum edge amplitude values within this region, which serves as an indicator of fringe clarity.

(2) $$C\left(\theta \right)=A_{\max}\left(\theta \right)-A_{\min}\left(\theta \right)$$

where C(θ) represents the contrast of the ampoule body region when the analyzer is rotated to angle θ, and $A_{\max}\left(\theta \right)$ and $A_{\min}\left(\theta \right)$ are the maximum and minimum edge amplitudes detected within that region, respectively.

By scanning through all angles, a contrast dataset is established:

(3) {C(θ)|θ=0°,1°,2°,…,90°,91°,…,180°}

Finally, the angle with the highest contrast is selected as the position where the fringes are the clearest, that is:

(4) $$\theta_{\max}=\arg\max_{\theta }C\left(\theta \right)$$

2. Stress calculation

According to the Jones algorithm, the light intensity *I* in this experimental system can be calculated as:

(5) $$I=\left(A_{x}^{2}+A_{y}^{2}+2A_{x}A_{y}\exp\left(i\delta_{x}-i\delta_{y}\right)+\exp\left(i\delta_{x}-i\delta_{y}\right)\right)\left(1-\cos(\varphi -2\theta )\right)$$

where $A_{x}$ and $A_{y}$ are the amplitude components of the incident light in directions *x* and *y*, and $\delta_{x}$ and $\delta_{y}$ are the phase delays in directions *x* and *y*, respectively.

According to Equation (5), when the phase difference is φ=2θ, the light intensity becomes zero. The optical path difference Δ is proportional to the phase difference φ, so when the analyzer is rotated by θ, the optical path difference Δ can be calculated using the following formula:

(6) $$\Delta =\frac{\lambda }{\pi }\cdot \theta ,\;\pi =180{}^{\circ}$$

where Δ represents the optical path difference, θ is the rotation angle of the analyzer, and λ is the wavelength of the incident light. It should be noted that due to the periodic nature of polarization, when θ exceeds 90°, the equivalent analysis angle of 180°−θ should be used instead.

Based on the acquired analyzer rotation angle θ, the optical path difference Δ is calculated. Then, using the material’s photoelastic constant *C* and the ampoule body thickness *h*, the stress value in the ampoule body can be determined as follows:

(7) $$\sigma =\frac{\Delta }{C\cdot h}$$

### 3.3. Experimental Results

**Figure 5:**
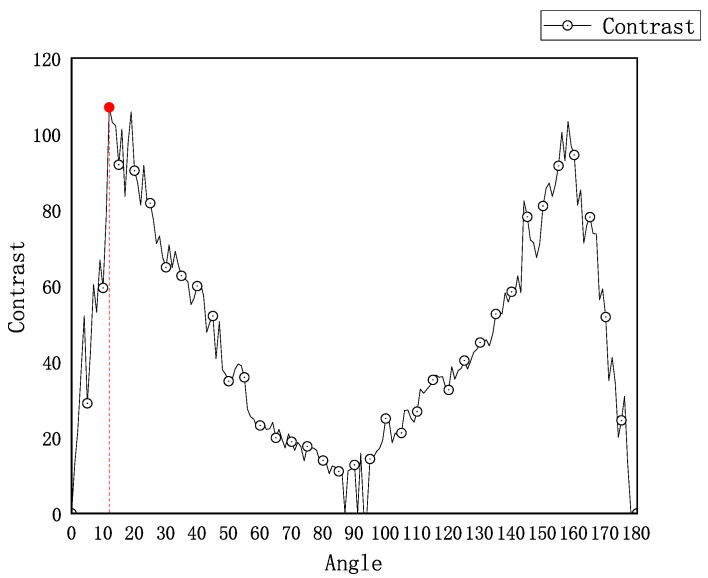
Edge contrast of the ampoule wall region as a function of polarizer angle. Maximum contrast at 12° indicates optimal fringe visibility, corresponding to an optical path difference of 37.68 nm and a stress sensitivity of 25.12 nm/mm.

In this experiment, the stress distribution of the glass ampoule was analyzed by measuring the variation in fringe edge amplitude contrast at different analyzer rotation angles in the ampoule wall region. Figure 5 shows the curve of edge amplitude contrast as a function of the analyzer angle. The experimental results indicate that the contrast of the fringe region varies periodically with the rotation of the analyzer, forming a complete cycle every 180°. As shown in Figure 5, during clockwise rotation, the edge amplitude contrast reaches its maximum when the analyzer angle is 12°, indicating the clearest interference fringes at this position. Based on this angle and using Equation (6), the optical path difference is calculated to be 37.68 nm. Since the light passes through the ampoule wall twice, the effective optical path length in the calculation is considered as twice the wall thickness, resulting in a unit optical path difference of approximately 25.12 nm/mm. It should be noted that this stress value corresponds to a specific measurement region of a single ampoule and serves as an example to illustrate the stress measurement method.

To verify the repeatability of the system, the same ampoule was measured ten times under fixed conditions. The results show that the unit optical path difference remained stable within the range of 23.12–27.12 nm/mm, with a fluctuation of approximately ±2 nm/mm, indicating good repeatability. In addition, given the system’s angular resolution of 1° and based on the Senarmont compensation principle (Equation (6)), the theoretical resolution of the optical path difference measurement is estimated to be about ±3nm, which can be regarded as the measurement accuracy of the system.

To further evaluate the reliability and feasibility of the proposed method, four batches of ampoule samples were selected for comparison experiments. Each sample was tested using both a commercial polariscope (model: YLY-03S; Jinan Sumspring Experimental Instrument Co., Ltd., Jinan, China) and the stress detection system developed in this study. Both methods performed five repeated measurements per sample, and the mean and standard deviation were calculated. The results are shown in Table 1.

Table 1 shows that the average optical path difference results obtained by the two methods are highly consistent, with the maximum deviation not exceeding 1 nm. This indicates that the proposed method achieves measurement accuracy comparable to that of the commercial polariscope. It should be noted that the minimum rotation step of the commercial instrument is 0.1°, while that of the proposed system is 1°. Therefore, the measurement fluctuation of the proposed method is slightly larger, resulting in a relatively higher standard deviation. Nevertheless, the results remain within a reasonable range, demonstrating that the system provides good measurement consistency and practical feasibility.

In addition, the measurement of commercial polariscopes relies on the operator’s subjective observation and experience in interpreting interference fringes, making the process complex and susceptible to human error. In contrast, the proposed method automatically acquires a sequence of polarized images and determines the optimal analyzer angle based on a contrast analysis algorithm, enabling fully automated stress calculation. This approach significantly reduces manual intervention and subjective bias. While maintaining high accuracy, it also offers simple operation, fast detection speed, and a high degree of automation, making it especially suitable for online inspection of glass containers in production lines.

In summary, although the proposed system has a lower angular resolution (1°), it can still achieve stress measurement results that closely match those obtained by the commercial polariscope without any manual intervention. These results confirm the feasibility and engineering potential of the proposed method for stress detection in glass ampoules.

In addition, random inspections were conducted on ampoules from different batches. Some samples showed optical path difference per unit thickness significantly higher than the 40 nm/mm limit specified in the Neutral Borosilicate Glass Ampoule standard (YBB00322005-2-2015) [30]. Analysis indicates two main reasons for this deviation: (1) the tested ampoules may have relatively high residual stress from the manufacturing process; (2) a general industrial camera was used in the experiment, which may have limited polarization accuracy. To address these issues, higher-precision polarization devices or an optical microscope compensation system will be considered for future validation.

## 4. Dimensional Measurement

### 4.1. Measurement Principle

The dimensional measurement in this study is based on computer vision technology, aiming to accurately measure the key geometric parameters of ampoules. The core idea is to capture high-resolution images of the ampoules using an industrial camera, and then apply advanced image processing algorithms together with calibrated camera parameters to convert pixel coordinates in the image into actual physical dimensions. This method can reliably identify and reject ampoules with abnormal dimensions, ensuring product quality and production consistency.

In the dimensional measurement process, camera calibration is a critical step. Its main purpose is to establish an accurate mapping between the image coordinate system and the real-world coordinate system [31]. Common calibration tools include dot arrays or standard checkerboard calibration plates. By processing calibration images using Halcon software (Version 24.11), the system can precisely estimate the camera’s internal parameters (such as focal length, principal point position, and distortion coefficients) and external parameters (camera position and orientation in space) [32]. After calibration, the system can measure target dimensions accurately at the pixel level and convert pixel distances into real-world physical sizes based on the calibration parameters, ensuring accuracy, repeatability, and robustness of the measurement results.

The ultimate goal of dimensional measurement is to obtain the true physical dimensions of the ampoule. Using the scale factor between pixels and physical units obtained from camera calibration, the actual physical size of each part can be calculated. The dimension conversion formula is as follows:

(8) $$D_{\mathrm{real}}=D_{\mathrm{pixel}}\cdot ScaleFactor$$

where $D_{\mathrm{pixel}}$ is the pixel size and ScaleFactor is the scale factor. The scale factor ScaleFactor is obtained through camera calibration using Halcon software, and its calculation formula is as follows:

(9) $$ScaleFactor=\frac{PixelSize}{magnification}$$

where PixelSize is the pixel size (the width of a single pixel is used for horizontal calculations, and the height of a single pixel is used for vertical calculations), and magnification is the magnification of the lens. According to the calibration results in Section 4.2.1, the pixel sizes in the vertical and horizontal directions are 3.44929 μm and 3.45 μm, respectively. With an magnification of 0.0880748, the corresponding scale factors are 39.15 μm/pixel (vertical) and 39.16 μm/pixel (horizontal). These calibrated scale factors are applied in the subsequent dimensional calculations to ensure measurement accuracy.

### 4.2. Method

**Figure 6:**
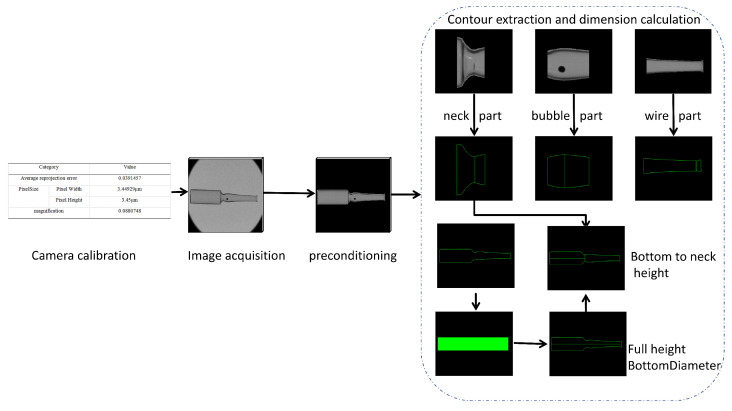
Workflow of dimension Inspection Process.

To clearly present the overall process of dimensional measurement, this paper first provides a flowchart of the ampoule dimensional measurement method, as shown in Figure 6. The process includes camera calibration, image acquisition, image preprocessing, contour extraction, and dimension calculation. This section will explain the implementation of each step in detail based on this flow.

#### 4.2.1. Camera Calibration

First, the dot array calibration board is placed on the experimental table, and the camera position is adjusted so that the calibration board is fully within the field of view, occupying about one-quarter to one-third of the view. Then, multiple sets of images covering the entire field of view are captured by changing the position and angle of the calibration board several times. The calibration images are processed using the calibration assistant in Halcon software. After selecting valid images, the system automatically estimates the camera’s internal and external parameters and corrects geometric distortion, ultimately generating a calibration parameter file [31]. As shown in Table 2, the calibration results indicate an average reprojection error of 0.0391, which meets the measurement accuracy requirements.

#### 4.2.2. Image Acquisition

**Figure 7:**
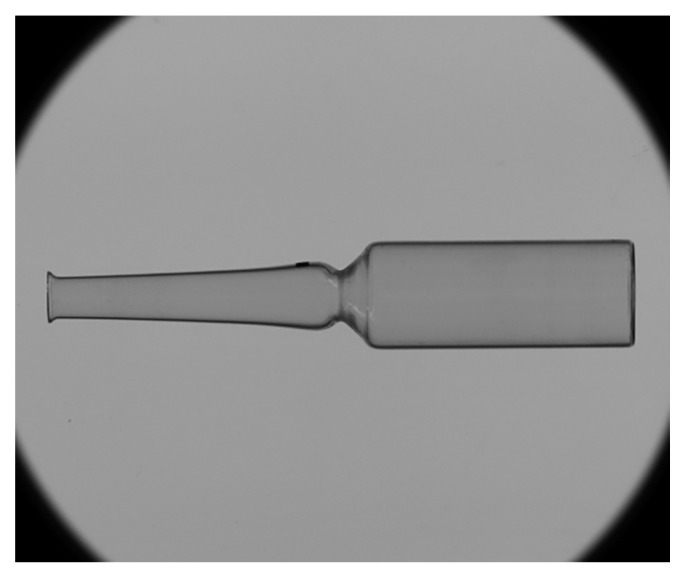
Raw image of the ampoule captured under telecentric backlight illumination.

Camera calibration, as an initialization step of the system, is performed only once before the inspection starts. After calibration is completed, it does not need to be repeated during subsequent image acquisition. During actual inspection, ampoules are conveyed one by one into the dimensional and defect detection area. The camera captures high-resolution images at the moment each ampoule passes through (see Figure 7), providing the data foundation for subsequent image processing.

#### 4.2.3. Image Preprocessing

**Figure 8:**
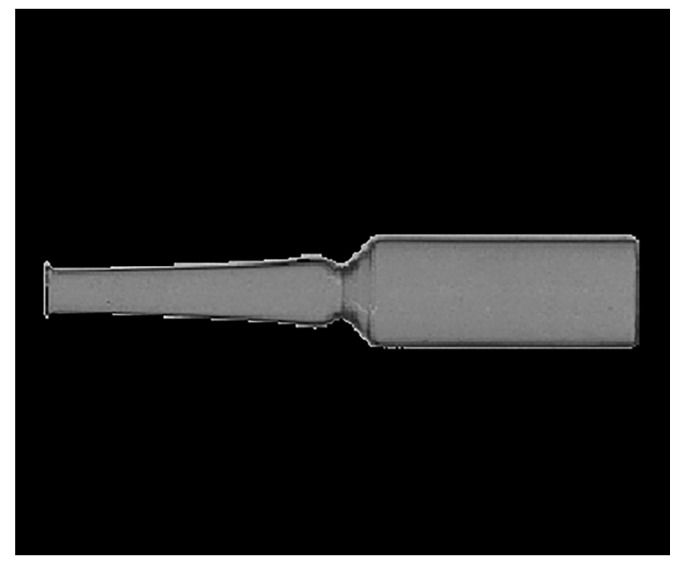
Preprocessed ampoule image.

To improve measurement accuracy, the captured ampoule images are first converted to grayscale, followed by histogram equalization to enhance image contrast. Then, techniques such as Gaussian filtering are applied to remove noise while preserving edge details. Binary thresholding is used to extract the ampoule region and eliminate background interference. Finally, the extracted ampoule region is rotation-corrected to ensure it is displayed horizontally, as shown in Figure 8.

#### 4.2.4. Contour Extraction and Size Calculation

**Figure 9:**
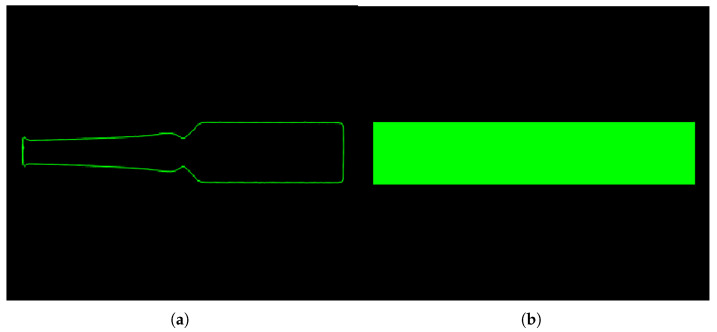
Fitting the minimum circumscribed rectangle. (**a**) The sub-pixel outline of the ampoule bottle, (**b**) Minimum Bounding Rectangle.

For the preprocessed ampoule images, a subpixel edge detection algorithm is applied to extract the contours of the ampoules, improving measurement accuracy. The extracted subpixel contours are then fitted with a minimum bounding rectangle (see Figure 9) to calculate the overall height of the ampoule (long side of the rectangle) and the base outer diameter (short side of the rectangle).

**Figure 10:**
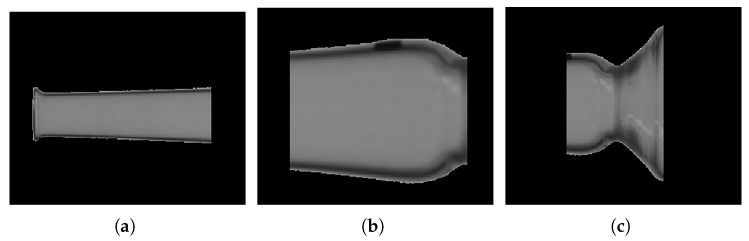
Key dimensional zones of the ampoule: (**a**) stem, (**b**) bulb, and (**c**) neck.

To clearly present the dimensional parameters measured in the upper part of the ampoule, three regions of interest (ROIs) are defined on the horizontally aligned ampoule (see Figure 10). These ROIs correspond to the following parameters:

- Stem outer diameter;
- Bulb outer diameter;
- Neck outer diameter.

Since the boundaries of the extracted ampoule region may not be clearly defined, grayscale equalization and Gaussian blur are applied to enhance edge clarity. Subpixel edge detection is then performed within each to extract complete, closed contours. Finally, vertical projection is used to calculate the stem outer diameter (minimum projection value), neck outer diameter (minimum projection value), and bulb outer diameter (maximum projection value). With the base and neck outer diameters known, the height from base to neck can be computed as the Euclidean distance between the center points of the base and neck diameters.

#### 4.2.5. Size Conversion

Using the above method, the pixel distances of the six key dimensions of the ampoule can be calculated. Based on the calibrated pixel width and height, along with the camera magnification, the pixel values are converted into actual physical dimensions using Equations (8) and (9).

### 4.3. Experimental Comparison and Result Analysis

#### 4.3.1. Experimental Design and Statistical Metrics

To evaluate the measurement accuracy and stability of the dimensional inspection system, 15 randomly selected ampoules were used as test samples. Two reference methods—caliper measurement and pixel contour extraction combined with curve fitting—were employed for comparison.

To ensure data reliability, each method performed five repeated measurements for each sample. All measurements were conducted under the same lighting conditions and camera settings. The average of the five measurements was taken as the final dimension value, and the standard deviation (SD) was used as an indicator of repeatability.

The error and standard deviation of error were computed using the following equations:

Measurement error:

(10) $$e_{i}=m_{i}-t_{i}$$

Average error:

(11) $$\bar{e}=\frac{1}{N}\sum_{i=1}^{N}e_{i}$$

Standard deviation of error:

(12) $$SD=\sqrt{\frac{1}{N-1}\sum_{i=1}^{N}{\left(e_{i}-\bar{e}\right)}^{2}}$$

Here, $m_{i}$ represents the measured value, $t_{i}$ is the caliper reference value, and *N* denotes the number of samples.

For the five repeated measurements of each sample, the within-sample standard deviation is defined as follows:

(13) $$SD_{i}=\sqrt{\frac{1}{n-1}\sum_{j=1}^{n}{\left(m_{ij}-\bar{m_{i}}\right)}^{2}}$$

Here, n=5 represents the number of repeated measurements, and $\bar{m_{i}}$ is the mean of the five measurements for the sample.

The overall repeatability is expressed as the average of the standard deviations across all samples:

(14) $$\bar{SD}=\frac{1}{N}\sum_{i=1}^{N}SD_{i}$$

#### 4.3.2. Comparison of Dimensions with Known Caliper Values

Since the stem outer diameter, bulb outer diameter, and neck outer diameter cannot be directly measured with calipers, caliper measurements were primarily performed for the overall height, base outer diameter, and height from base to neck. Comparative experiments were conducted using two methods, with the caliper measurements serving as reference values:

- Method 1: Pixel contour extraction combined with curve fitting. In this method, the ampoule contours are extracted at pixel-level resolution, and linear or polynomial curve fitting is applied to calculate key dimensions that cannot be directly measured by calipers. This method serves as a baseline image-based measurement technique.
- Method 2: Subpixel contour extraction combined with vertical projection (our method). Our method extracts contours at subpixel accuracy, improving edge precision. Vertical projection is applied along the extracted contours to calculate the stem, bulb, and neck diameters, effectively reducing measurement errors compared with Method 1.

To evaluate overall accuracy, three representative dimensions—overall height, base outer diameter, and height from base to neck—were measured using both methods. The mean error, mean absolute error, and standard deviation of the error were calculated for each method. The results are presented in Table 3, clearly demonstrating the improved performance of our method.

As shown in Table 3, the mean measurement errors of both methods are within ±0.2 mm, indicating high accuracy of the system. Method 2 exhibits slightly lower mean absolute errors than Method 1 for overall height and base outer diameter, demonstrating that subpixel contour extraction combined with the vertical projection algorithm effectively reduces pixelation errors. For height from base to neck, Method 2 shows a lower standard deviation than Method 1, indicating better repeatability. Considering all three dimensions, Method 2 achieves mean errors closer to zero and superior stability.

#### 4.3.3. Dimension Comparison Without Using Calipers (True Value)

Since the stem outer diameter, bulb outer diameter, and neck outer diameter cannot be directly measured with calipers, the standard deviation of five repeated measurements for each method was used as an indicator of consistency. Table 4 lists the mean ± standard deviation for each sample, and the summary statistics reflect the stability differences between the two methods.

Based on Table 4, Method 2 shows slightly lower standard deviations than Method 1 for all three dimensions, indicating that the subpixel contour extraction combined with vertical projection exhibits higher stability under conditions such as edge discontinuities and lighting fluctuations. This method reduces quantization errors through subpixel edge localization and mitigates the impact of local noise via vertical projection computation.

#### 4.3.4. Discussion and Analysis of Results

Based on the experimental results, the proposed dimensional measurement method combining subpixel contour extraction with vertical projection outperforms the traditional pixel contour plus curve fitting method in both accuracy and stability. For the three dimensions that can be verified with calipers—overall height, base outer diameter, and height from base to neck—the mean errors of the proposed method are all within ±0.2 mm, with relatively small standard deviations, resulting in more concentrated and stable measurements. In contrast, the traditional method exhibits noticeable fluctuations for some samples. The proposed approach effectively reduces measurement deviations caused by local anomalies such as reflections and edge discontinuities through subpixel edge localization and vertical projection computation. Although the error of individual samples is relatively large, the overall measurement still maintains high consistency and reliability. These results indicate that the proposed method achieves precise dimensional measurements while maintaining good repeatability and robustness, meeting the requirements for high stability and accuracy in ampoule production inspection.

## 5. Defect Detection

### 5.1. Measurement Principle

Defect detection is a critical step in the quality control of ampoules. Its main goal is to automatically identify and classify potential surface defects using advanced visual inspection technologies. Common defects on ampoule surfaces include stains, and gas lines, all of which can significantly affect product quality and safety. Traditional inspection methods mainly rely on manual visual checks, where trained personnel inspect the ampoule surfaces for defects. However, manual inspection is highly subjective and easily influenced by human factors, resulting in inconsistent outcomes and a higher risk of false detections or missed defects [33].

With the development of computer vision technology, some ampoule manufacturers have started using machine vision systems based on advanced algorithms for automatic inspection. However, when facing strict industry quality standards, these systems still have limitations in terms of efficiency, detection accuracy, and real-time performance. As a result, deep learning-based visual inspection methods have gradually become an important approach to ensuring ampoule quality, showing clear advantages in both detection accuracy and system adaptability.

Deep learning-based defect detection methods can be mainly divided into two categories: unsupervised learning and supervised learning. Unsupervised methods typically learn the features of normal samples (excluding defects) to build a normal pattern model, and detect potential defects by identifying deviations from this model in new samples. In contrast, supervised methods rely on the powerful feature extraction and learning capabilities of deep neural networks, using large amounts of labeled defect samples for training to achieve accurate identification and localization of defect regions on ampoules [34].

In recent years, the YOLO (You Only Look Once) series of supervised object detection models has gained widespread attention in the field of visual inspection. YOLO employs a single-stage, end-to-end detection framework that directly predicts object classes and their locations from input images, achieving a good balance between detection accuracy and computational efficiency [35]. YOLOv8 [36] is a relatively complete and widely used version within the YOLO series, with optimizations in detection accuracy, robustness, and inference speed. Compared with other object detection algorithms, YOLOv8 offers notable advantages in processing speed and computational efficiency, making it particularly suitable for real-time inspection applications in industrial production.

The multi-scale detection capability of YOLOv8 enables it to effectively recognize defect features at different image scales, which is especially important for detecting small defects on ampoules. In addition, YOLOv8 uses advanced feature fusion techniques that help maintain high detection accuracy even under challenging background conditions, such as reflections or shadows. In real production environments, YOLOv8 demonstrates strong robustness and stability, providing reliable technical support for ampoule defect inspection. Therefore, this study adopts YOLOv8 as the core model for detecting surface defects on ampoules.

### 5.2. Method

#### 5.2.1. Dataset Preparation

To train the YOLOv8 model for detecting surface defects on ampoules, it is first necessary to build an annotated dataset that includes various types of defects. According to the Appearance Defect Evaluation Guide for Pharmaceutical Glass Containers issued by the China Pharmaceutical Packaging Association [37], and based on quality requirements from ampoule manufacturers, this study classifies ampoule surface defects into four categories: bumpcheck (impact damage), check (surface cracks), stain (contamination), and gasline (air lines).

**Figure 11:**
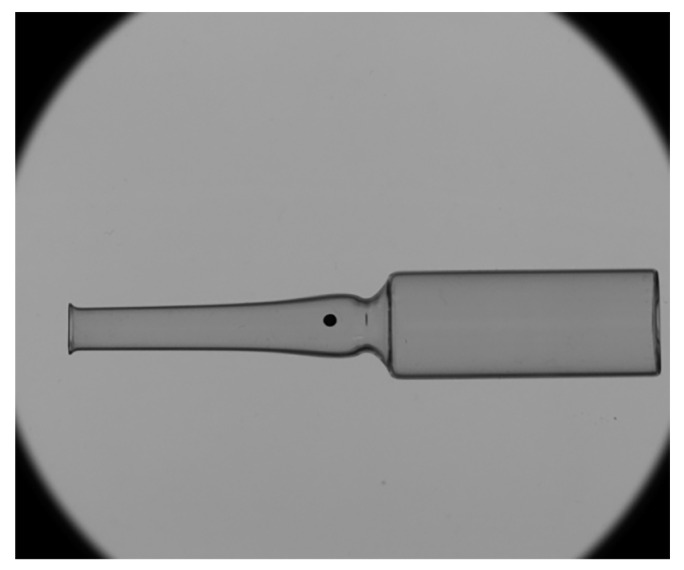
Image of the original ampoule.

**Figure 12:**
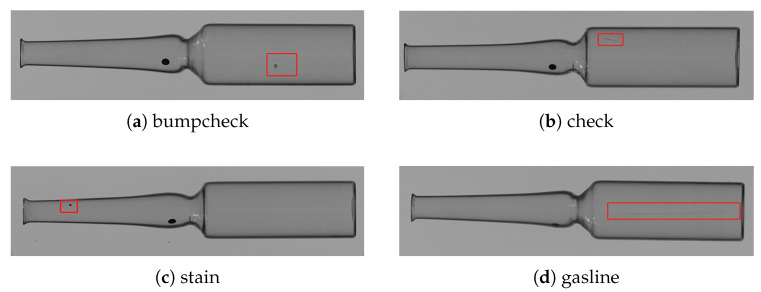
Images of four types of defect samples: (**a**) bumpcheck; (**b**) check; (**c**) stain; and (**d**) gasline.

During dataset construction, ampoules with different types of surface defects were selected, and defect images were captured using the defect detection system developed in this study. The original resolution of the collected images was 2456 × 2052, as shown in Figure 11. To reduce computational load and improve training efficiency, the images were resampled to a size of 1024 × 1024. Figure 12 shows sample images of four defect types, with red boxes indicating their locations. The defects are defined as follows:

- Bumpcheck: Crescent-shaped or dot-like surface marks caused by mechanical impact or contact between glass containers.
- Check: Non-penetrating fine cracks on the glass surface, typically short but relatively deep, resulting from thermal or mechanical stress.
- Stain: Contaminants deposited, adhered, or embedded on the inner or outer surfaces of the container that cannot be removed.
- Gasline: Tiny, elongated bubbles within the glass cross-section.

Relying solely on the original captured images is not sufficient to meet the training requirements of deep learning models, especially when defect samples on ampoule surfaces are limited. In such cases, data augmentation plays a key role in improving the model’s generalization ability. By introducing diverse image transformations, the model can learn more robust features and reduce its dependence on specific backgrounds, viewing angles, or lighting conditions.

During data acquisition, approximately 60 ampoule samples exhibiting genuine surface defects were selected. Because some samples contained multiple defect regions or more than one defect type, and to ensure sufficient capture of surface details, each sample was imaged from multiple viewpoints. In total, 120 real defect images were obtained, comprising 32 bumpcheck, 36 check, 30 gasline, and 34 stain instances, resulting in 132 annotated defect occurrences.

In this study, the Augmentor [38] data augmentation library was used to expand the original dataset. The augmentation methods include operations such as rotation, scaling, and brightness/contrast adjustment. Specifically, some images were randomly rotated to generate defect samples at different angles, helping the model learn how defects appear from multiple viewpoints. Other images were scaled to improve the model’s adaptability to ampoules of varying sizes. Additionally, by adjusting brightness and contrast, the model’s detection performance under complex lighting conditions was enhanced.

After data augmentation, the dataset was expanded to a total of 1200 images (including normal samples), with each image potentially containing one or more types of defects. The distribution of each defect type in the augmented dataset is shown in Table 5.

After data augmentation, the dataset needs to be labeled. However, labeling a large number of images is challenging. Manual labeling is time-consuming and labor-intensive, and prone to inconsistency due to subjective human judgment—especially when dealing with diverse defect types. To address this, this study proposes an efficient data annotation method that combines manual labeling of a small dataset with an automatic labeling tool. Specifically, a small dataset called littledata was first created, containing 120 images and five types of defects. The composition of littledata is shown in Table 6.

The defect regions in the small dataset littledata were manually annotated using the labeling tool LabelImg [39]. Each defect region was marked with a bounding box and assigned a corresponding defect category (such as bumpcheck, check, or stain). In this study, the annotations followed the YOLO dataset format, where each line includes the object class, the coordinates of the bounding box center, as well as its width and height.

After the small dataset littledata is labeled, it is used to perform an initial training of the YOLOv8 model. This process generates a set of initial YOLOv8 weights, which show good performance on the littledata dataset. Then, the weights trained on the small dataset littledata are used in combination with the X-AnyLabel [40] tool to automatically annotate the complete dataset. X-AnyLabel applies model inference to generate bounding boxes and class predictions for defects, and automatically outputs annotation files in YOLO format. To improve annotation quality, all automatically generated labels were manually verified and corrected to ensure the accuracy of both bounding boxes and class information. This method significantly reduced manual labeling time and improved the consistency and precision of annotations with the help of model-assisted labeling. It not only provided a high-quality data foundation for defect detection but also offered a scalable and efficient labeling solution for similar tasks.

#### 5.2.2. Model Training

The defect detection model was trained using an NVIDIA GeForce RTX 4070 GPU and an Intel(R) Core(TM) i5-14600KF CPU. To improve training stability and efficiency, a warm-up strategy was applied during the first three epochs, and automatic mixed precision (AMP) was enabled throughout training. To enhance the model’s generalization ability, various data augmentation techniques were applied to the input images, including scaling, translation, horizontal flipping, color jittering, and Mosaic augmentation (combining four images) in the early stages of training, which was disabled after the 10th epoch to allow the model to focus on real defect detection. The detailed training configurations are listed in Table 7.

#### 5.2.3. Evaluation Index

The experiment used precision (*P*), recall (*R*), and mean average precision (mAP) as evaluation metrics to assess the model’s detection performance on glass ampoules. The calculations for precision and recall are as follows:

(15) $$P=\frac{TP}{TP+FP}$$

(16) $$R=\frac{TP}{TP+FN}$$

In the formulas, TP represents the number of true positive detections correctly identified by the model; FP refers to the number of false positives, where background is mistakenly detected as a target class; and FN denotes the number of false negatives, where the model fails to detect target objects and classifies them as background.

In defect detection tasks, Average Precision (AP) is used to evaluate the detection performance for each individual class. The mean Average Precision (mAP) represents the average value of AP across all classes. AP is calculated by measuring the area under the Precision-Recall curve. Following the COCO evaluation standard [41], the all-point interpolation method is used. This method considers all recall values, and for each recall value *r*, it finds the maximum precision among all recall values greater than or equal to *r*. The final result is the area under the curve formed by these points.

(17) $$AP=\sum_{i=1}^{n}\left(R_{i}-R_{i-1}\right)\cdot \max_{\tilde{r}:\tilde{r}\geq R_{i}}P\left(\tilde{r}\right)$$

where $R_{i}$ is the *i* recall point, and $P(\tilde{r})$ is the maximum precision corresponding to recall value $\tilde{r}$.

The mean across all defect categories AP is then calculated as:

(18) $$mAP=\frac{1}{N}\sum_{i=1}^{N}AP_{i}$$

where *N* represents the total number of defect categories.

mAP@0.5 refers to mAP calculated when the IOU threshold is fixed at 0.5. mAP@0.5:0.95 represents the average of all mAP values when the IOU threshold ranges from 0.5 to 0.95 with a step size of 0.05, as shown below:

(19) $$mAP_{50-95}=\frac{1}{10}\sum_{t=0.5}^{0.95}mAP_{t}$$

Here, *t* represents different threshold values of IOU. This metric follows the COCO evaluation standard [41], which provides a more comprehensive assessment of the model’s detection performance.

### 5.3. Experimental Results

#### 5.3.1. Performance of the Proposed YOLO-Based Defect Detector

**Figure 13:**
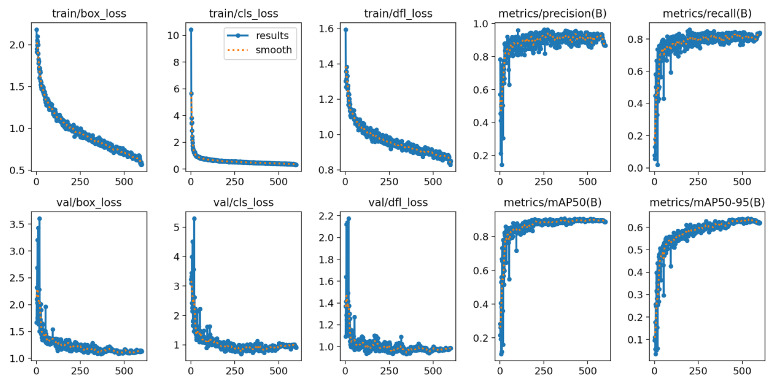
Training and validation curves of the YOLOv8 model, including loss, precision, recall, mAP@0.5, and mAP@0.5:0.95.

Figure 13 presents the training and validation curves of the box loss, classification loss, and DFL loss, together with the validation precision, recall, mAP@0.5 (corresponding to “mAP50” shown in the figure), and mAP@0.5:0.95. The training losses decrease smoothly and converge as the number of epochs increases. The validation losses exhibit similar downward trends without noticeable divergence from the training curves, suggesting that the model maintains stable performance on unseen data and does not show clear overfitting.The validation precision, recall, mAP@0.5, and mAP@0.5:0.95 curves increase steadily and begin to stabilize around 200–250 epochs, indicating that the network gradually improves its localization and classification capability as training proceeds. These results demonstrate that the model progressively refines the predicted bounding boxes and reliably distinguishes different defect categories based on consistent trends observed in both the training and validation sets.

Table 8 presents the main performance metrics of the model on the validation set for each defect category. As shown in the table, the model achieves an mean average precision (mAP) of 0.903 across all categories, indicating a high level of accuracy in defect detection.

**Figure 14:**
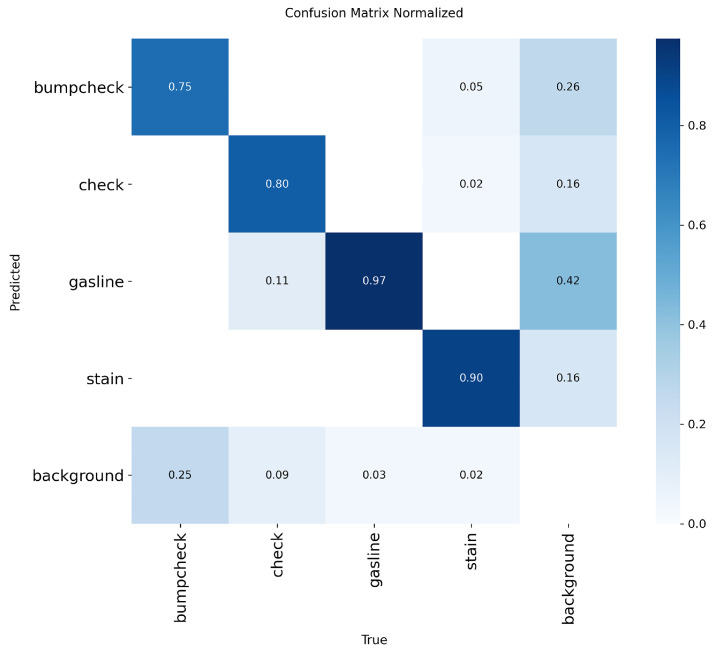
Confusion matrix of the YOLOv8n model on the validation dataset.

**Figure 15:**
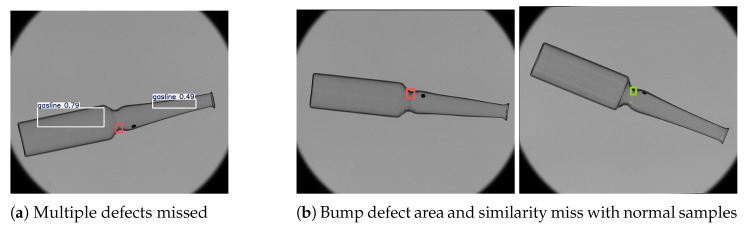
Two common types of missed detections in leak inspection. Red boxes mark undetected bumpcheck defects. (**a**) White boxes indicate defects that were successfully detected; (**b**) left shows a defective sample, right a normal sample with a green box highlights a bumpcheck-like necking defect.

Although the overall accuracy is satisfactory, the performance differs among individual defect types, and the bumpcheck category shows a notably lower recall (0.631). To better understand this issue, we examined the confusion matrix of the validation results(Figure 14). Approximately 25% of bumpcheck samples were predicted as background, which is the main reason for the reduced recall. Most of these missed cases occur in images containing multiple defects simultaneously. The high false-negative rate for bumpcheck in multi-defect images is mainly because the defect is small, has weak texture, and is often located near more prominent defects, which causes visual interference and makes the model more likely to overlook it or classify it as background. For example, when gasline defects and a bumpcheck appear together, the model tends to detect the gasline defects but may overlook the bumpcheck. Another common situation is that the missed bumpcheck defects are located near the neck of the ampoule, where regular scale marks and fine textures resemble small bumpcheck patterns. This visual similarity can cause the model to regard the defect as part of the normal structure, resulting in false negatives. A typical missed bumpcheck case is illustrated in Figure 15.

**Figure 16:**
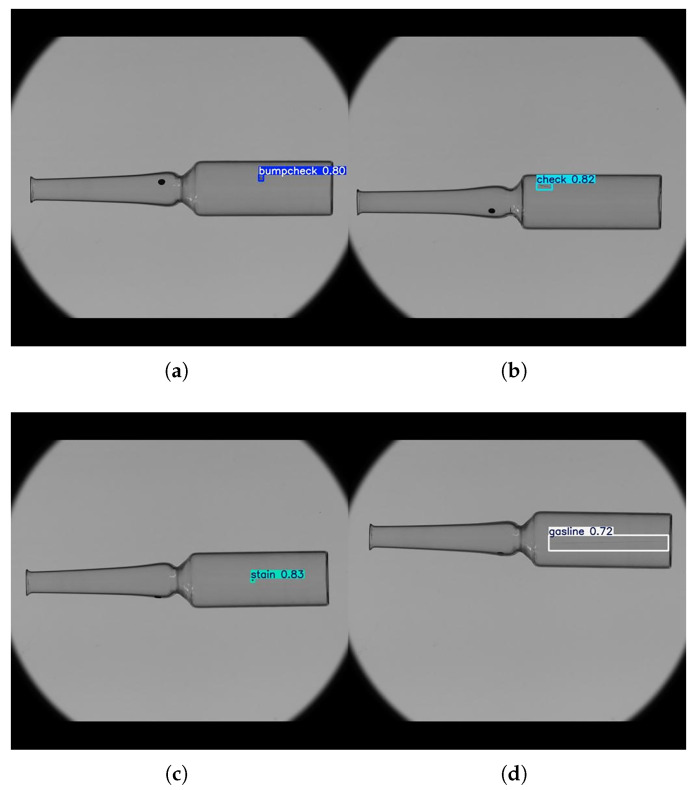
Defect detection and classification results of glass ampoules, (**a**) bumpcheck, (**b**) check, (**c**) stain, (**d**) gasline.

The recall and precision for the other defect categories (check, stain, and gasline) remain relatively high, indicating that the model performs consistently well on defects with stronger or more distinctive features. Despite the limitations on small bumpcheck defects in complex scenarios, the model performs reliably in most standard conditions. As shown in Figure 16, it successfully detects and classifies all defect types under varying illumination and background conditions, suggesting that the system can reliably detect critical defects in typical production-line environments.Based on these observations, several improvements could be explored in future work, such as increasing annotated samples for difficult regions, applying targeted data augmentation, or incorporating modules to enhance small-object detection. These refinements may further reduce false negatives and improve the system’s reliability compared with other detection models.

#### 5.3.2. Cross-Validation Evaluation

To assess the generalization capability of the YOLOv8-based defect detection model, a five-fold cross-validation was conducted on the annotated ampoule defect dataset. The dataset was randomly divided into five subsets, with four subsets used for training and the remaining one used for testing in each iteration. The average performance metrics obtained from the five validation rounds are summarized in Table 9.

As shown in Table 9, the model achieves consistently high values of Precision, Recall, and mAP@0.5 across all five folds, with only minor variations. This indicates stable performance under different data partitions and demonstrates strong generalization capability. Compared with the results obtained from a single validation set, the average performance in the cross-validation setting is slightly higher. This observation is reasonable for two reasons: first, cross-validation exposes the model to more diverse combinations of training data across multiple iterations, which enhances its overall fitting ability; second, several challenging defect samples contained in the original validation set are redistributed among different folds, reducing the bias associated with evaluating on a single, fixed subset. Overall, the five-fold cross-validation offers a more reliable and comprehensive reflection of the model’s performance in practical application scenarios.

#### 5.3.3. Robustness Analysis Under Common Industrial Disturbances

To evaluate the stability of the model under real production conditions, robustness tests were conducted using five types of image perturbations commonly encountered in industrial vision systems. Each perturbation was designed to simulate typical sources of interference on the production line, including:

- motion blur: simulates conveyor speed fluctuations or minor vibrations during camera exposure;
- brightness_contrast: reflects variations in lighting or contrast caused by unstable illumination, shadows, or changes in ambient brightness;
- jpeg_compression: mimics quality degradation resulting from image storage or compression during transmission;
- rotate_shift_scale: simulates slight pose variations of ampoules due to wobbling or inconsistent orientation on the conveyor;
- hue_saturation: represents color shifts caused by light source color temperature drift, LED aging, or similar effects.

The perturbation strength for each type was set to remain realistic and consistent with actual industrial conditions. After applying these perturbations, the Precision, Recall, and mAP@0.5 metrics were recalculated on the modified dataset. The results are summarized in Table 10.

The test results indicate that the model’s performance exhibits only minor changes under the five types of typical industrial perturbations. Compared with the baseline (P = 0.9330, R = 0.7970, mAP@0.5 = 0.9030), the variation of each metric is controlled within ±2.5%, demonstrating the model’s strong stability under challenging imaging conditions. Among the perturbations, motion blur has the most noticeable impact, with Precision and mAP@0.5 decreasing by 2.46% and 0.99%, respectively, mainly because blur reduces the clarity of defect edges; however, the decline is limited, indicating that the model can tolerate mild blurring. It is worth noting that Recall slightly increases under certain perturbations, as the weakening of background textures encourages the model to generate more low-confidence candidate boxes, reducing missed detections but slightly increasing false positives. In comparison, brightness/contrast variations, JPEG compression, and hue/saturation shifts have minimal impact on the model, with changes in mAP@0.5 below 1%, indicating strong adaptability to lighting fluctuations, compression artifacts, and color deviations. Rotation, translation, and scaling induce a small performance decline of approximately 1.23%, but the overall performance remains stable. Overall, the YOLOv8n defect detection model demonstrates robust performance under various typical industrial disturbances, maintaining reliable detection accuracy in practical production environments.

#### 5.3.4. Comparative Study with Other Detection Models

To select an appropriate defect detection framework, this study analyzed the characteristics of two-stage and single-stage detectors in industrial scenarios. Previous studies have shown that two-stage methods [42], such as Faster R-CNN [43], typically achieve 75–80% accuracy on standard industrial datasets like NEU-DET, but their inference speed is relatively slow and computationally demanding, which limits their applicability in real-time production environments. In contrast, single-stage detectors of the YOLO family demonstrate a favorable balance between speed and accuracy across various industrial datasets, including steel, metal surfaces, and electronic components, making them more suitable for real-time defect inspection. Based on this analysis, the YOLO series was selected as the core detection framework in this study. Comparative experiments were conducted within the YOLO family, including YOLOv5 [44], YOLOv7 [45], YOLOv8 [36], and YOLOv11 [36], to evaluate the performance advantage of YOLOv8 under consistent experimental conditions. The results are summarized in Table 11.

A comprehensive analysis of Table 11 shows that the YOLOv8 model offers the most balanced performance across accuracy, robustness, and speed—key factors in practical ampoule defect inspection. Although YOLOv11 shows slightly higher single-threshold precision and recall, the differences (P: 0.935 vs. 0.933; R: 0.819 vs. 0.797) are minor and do not result in a meaningful reduction of missed detections. In real-world production, accurate localization under strict tolerance is often more critical than marginal improvements in detection probability. A correctly identified defect is still considered invalid if its location deviates beyond the predefined quality threshold. Thus, we emphasize the mAP@0.5:0.95 metric, which reflects the model’s robustness across multiple IoU thresholds and better captures performance on defects with diverse shapes, sizes, and ambiguous boundaries. YOLOv8 achieves the highest mAP@0.5:0.95 (0.639), confirming its superior localization consistency—crucial for reliable industrial decision-making.

In terms of efficiency, YOLOv8 reaches 233 FPS, the highest among all models tested, even outperforming YOLOv5 and YOLOv11. This speed advantage supports high-throughput inline inspection without adding processing delays. The inference was run on an NVIDIA RTX 4070 without TensorRT acceleration, as detailed in Section 5.2.2.

In summary, YOLOv8 offers the best combination of localization robustness, real-time speed, and deployment feasibility. While YOLOv11 achieves marginally higher P/R, YOLOv8’s superior mAP@0.5:0.95 and faster inference make it the most appropriate choice for the proposed multi-parameter inspection system.

## 6. Ablation Study

To evaluate the advantages of the proposed unified visual inspection platform in terms of overall performance and system design, design-level ablation experiments were conducted. Since it is challenging to construct two complete systems under practical conditions, a semi-quantitative comparison based on system parameters and camera specifications was employed. The integrated deployment scheme and the separate deployment scheme were analyzed and estimated in terms of hardware configuration, acquisition efficiency, and system synchronization complexity.

In conventional schemes, stress measurement, dimensional inspection, and defect detection are typically performed using separate optical paths and camera modules. In contrast, the proposed unified visual platform integrates the dimensional and defect detection modules into the same optical path and camera, achieving multi-task inspection solely through light source switching and algorithmic branching. To evaluate the engineering advantages of this integrated design, this paper selects five indicators for comparison: the number of cameras, light sources and imaging channels, image acquisition time (estimated based on camera frame rate), system synchronization difficulty, and calibration workload.

The camera used is the Basler acA2440-20gmLET (Sony IMX264 sensor, global shutter; Basler AG, Ahrensburg, Germany), a customized version of the Basler acA2440-20gm. Except for the resolution, it is identical to the standard model, with a typical frame rate of 22.7 fps (corresponding to a single-frame period of approximately 44.1 ms). In this study, both dimensional and defect inspections were performed using images acquired at the native resolution; any subsequent downsampling or ROI extraction was conducted only during algorithm processing and did not affect the acquisition rate. Therefore, the image acquisition time was estimated based on this frame rate.

As shown in Table 12, for the same inspection tasks, the integrated visual system outperforms the conventional separate scheme in terms of hardware count, optical structure, synchronization complexity, and cost. In particular, regarding image acquisition efficiency, the integrated system can reduce time delay by approximately 50%, providing a clear advantage for production line inspection or online quality control scenarios. It should be noted that this section presents a design-level ablation study, and the data are estimated based on camera specifications and engineering experience rather than measured from fully built systems. In future work, prototypes of both systems will be constructed to conduct experimental verification of acquisition delay, alignment errors, and algorithm fusion performance, providing quantitative evidence of the integrated system’s performance improvements in real production environments.

## 7. Discussion

Conventional inspection systems for transparent containers typically treat stress analysis, dimensional measurement, and defect detection as independent tasks, each requiring separate hardware modules and data processing pipelines. This separation not only increases system complexity and cost, but also limits opportunities for cross-task optimization and joint decision-making.

The platform proposed in this work represents a shift toward an integrated framework that combines stress, dimensional, and defect inspection into a coordinated system. By sharing imaging hardware and synchronizing data flows, the system achieves higher operational efficiency and facilitates multi-task reasoning. For example, critical defects near the bottle neck can be cross-referenced with local stress concentrations and dimensional deviations, enabling a composite quality judgment rather than relying on isolated criteria.

This fusion of sensory data streams lays the foundation for global modeling of container health status. In addition to identifying defective items, the system can generate structured data suitable for predictive quality assessment and risk evaluation. Such capabilities align with the vision of intelligent manufacturing, where inspection systems not only detect problems, but also anticipate failures and guide process optimization.

Future research will focus on enhancing this integration by refining the task scheduling logic, improving real-time throughput, and establishing correlation models between defect patterns and residual stress distributions. Ultimately, we aim to evolve the platform from a multi-parameter inspection tool into an intelligent system for failure prediction and quality forecasting.

Moreover, establishing a correlation model between surface defect patterns and residual stress distribution opens up new possibilities for intelligent inspection. Instead of performing stress analysis uniformly across the entire sample, the system can dynamically focus stress evaluation only on regions exhibiting critical defects, as detected by the vision module. This targeted inspection strategy can significantly reduce measurement time and computational load, while maintaining high diagnostic confidence.

By leveraging such cross-modal information coupling, the inspection process can shift from passive detection to active fault prediction, wherein localized stress concentrations around defects signal potential risks of fracture or leakage. This transition not only enhances the platform’s value in predictive maintenance scenarios but also contributes to improving overall system throughput and scalability in industrial deployments.

## 8. Conclusions

This study presents a unified vision-based inspection platform for transparent pharmaceutical containers, capable of simultaneously assessing residual stress, dimensional accuracy, and surface defects. Our main contributions can be summarized as follows:

- We designed a compact system architecture that integrates three inspection modules into a single vision pipeline, improving hardware utilization and reducing system complexity.
- A task scheduling mechanism was implemented to coordinate shared imaging resources, enabling fast and conflict-free execution of dimensional and defect inspections.
- A deployment strategy was developed for robust performance under variable lighting and bottle orientation, including deep learning-based defect detection and telecentric imaging-based dimension evaluation.

The proposed platform has been validated under production-like conditions, achieving a dimensional accuracy of ±0.2 mm, stress resolution of ±3 nm, and defect detection mAP of 90.3%. These results demonstrate the system’s potential for real-world deployment in pharmaceutical and packaging industries.

## Figures and Tables

**Table 1:** Comparison of measurement results of optical path difference between the method in this paper and commercial stress meters (mean ± standard deviation).

Sample Number	YLY-03S (nm)	Our Method (nm)	Difference (nm)
1	20.03 ± 0.67	20.24 ± 2.42	0.21
2	13.05 ± 0.32	13.26 ± 2.42	0.21
3	10.19 ± 0.99	11.16 ± 1.21	0.98
4	10.47 ± 0.21	11.16 ± 1.21	0.69

**Table 2:** Calibration results.

Category	Subcategory	Value
Average reprojection error	–	0.0391457
PixelSize	Pixel Width $S_{x}$	3.44929 μm
	Pixel Height $S_{y}$	3.45 μm
magnification	–	0.0880748

**Table 3:** Comparison of true value sizes.

Size Parameters	Method	Mean Error/mm	Mean Absolute Error/mm	Standard Deviation of Error/mm
Overall Height	Method 1	−0.054310667	0.068193333	0.070648641
	Method 2	−0.023569333	0.063708	0.077739731
Base outer diameter	Method 1	−0.017354667	0.086096	0.105348996
	Method 2	0.026893333	0.085477333	0.100795091
Height from base to neck	Method 1	0.024254667	0.192844	0.229908536
	Method 2	0.080270667	0.191494667	0.225058503

Note: Method 1 refers to pixel contour extraction combined with curve fitting, and Method 2 refers to subpixel contour extraction combined with vertical projection (i.e., the proposed method). Errors were calculated according to Equations (11) and (12).

**Table 4:** Comparison of true value and non-true value dimensions.

Number	Stem Outer Diameter (mm)		Bulb Outer Diameter (mm)		Neck Outer Diameter (mm)	
	Method 1	Method 2	Method 1	Method 2	Method 1	Method 2
1	6.19 ± 0.18	6.29 ± 0.03	10.14 ± 0.08	11.10 ± 0.08	8.12 ± 0.05	8.11 ± 0.05
2	6.43 ± 0.07	6.46 ± 0.04	9.75 ± 0.02	9.73 ± 0.03	8.15 ± 0.04	8.07 ± 0.07
3	6.20 ± 0.27	6.33 ± 0.05	10.17 ± 0.13	10.04 ± 0.07	8.42 ± 0.08	8.37 ± 0.07
4	6.40 ± 0.03	6.45 ± 0.02	10.47 ± 0.13	10.42 ± 0.07	7.82 ± 0.08	7.95 ± 0.05
5	6.36 ± 0.28	6.47 ± 0.03	10.70 ± 0.07	10.65 ± 0.12	9.51 ± 0.06	9.50 ± 0.05
6	6.50 ± 0.13	6.11 ± 0.19	10.85 ± 0.06	10.75 ± 0.05	9.29 ± 0.06	9.30 ± 0.05
7	6.28 ± 0.21	6.50 ± 0.03	10.50 ± 0.9	9.94 ± 0.15	9.24 ± 0.06	9.26 ± 0.08
8	6.08 ± 0.17	6.24 ± 0.10	10.77 ± 0.11	10.69 ± 0.07	9.35 ± 0.08	9.49 ± 0.09
9	5.98 ± 0.12	6.05 ± 0.04	11.25 ± 0.10	11.16 ± 0.06	9.44 ± 0.05	9.41 ± 0.07
10	5.94 ± 0.35	6.14 ± 0.06	10.75 ± 0.04	10.76 ± 0.05	9.42 ± 0.02	9.42 ± 0.04
11	5.97 ± 0.17	6.17 ± 0.08	11.30 ± 0.13	11.20 ± 0.14	9.39 ± 0.11	9.48 ± 0.08
12	6.18 ± 0.18	6.54 ± 0.14	10.23 ± 0.23	9.96 ± 0.24	9.36 ± 0.06	9.35 ± 0.05
13	6.19 ± 0.18	6.22 ± 0.11	10.70 ± 0.16	10.15 ± 0.13	9.35 ± 0.11	9.35 ± 0.09
14	6.02 ± 0.19	6.12 ± 0.04	11.11 ± 0.05	11.03 ± 0.04	9.35 ± 0.05	9.27 ± 0.06
15	6.35 ± 0.13	6.43 ± 0.12	10.44 ± 0.14	10.37 ± 0.14	9.43 ± 0.09	9.48 ± 0.02
**Mean SD**	0.176858298	**0.07238281**	0.102973133	**0.095109479**	0.065135133	**0.062129802**

Note: Each sample was measured five times, and ± indicates the within-sample standard deviation; bold values represent the better (more stable) results. The average SD was calculated according to Equations (13) and (14).

**Table 5:** Quantity of surface defects of type 4 ampoules.

Category	Bumpcheck	Check	Stain	Gasline
Number	256	292	243	278

**Table 6:** littledata dataset.

Category	Bumpcheck	Check	Stain	Gasline	Good	All
train	16	16	16	16	16	80
test	4	4	4	4	4	20
val	4	4	4	4	4	20

**Table 7:** Experimental Parameter settings.

Parameters	Setup
Epoch	600
Batch size	8
Learning rate	0.01
Weight decay	0.0005
Momentum	0.937
Image size	1024 × 1024
Optimizer	SGD
Patience	100

**Table 8:** Detection results of various categories of the YOLOv8n model.

Category	*P*	*R*	mAP@0.5	mAP@0.5:0.95
bumpcheck	0.889	0.631	0.803	0.556
check	0.955	0.774	0.874	0.614
stain	0.995	0.833	0.973	0.646
gasline	0.893	0.949	0.96	0.741
all	0.933	0.797	0.903	0.639

**Table 9:** Cross-validation results.

Fold	*P*	*R*	mAP@0.5
1	0.9359	0.8698	0.9325
2	0.9435	0.8634	0.9287
3	0.8685	0.8802	0.9173
4	0.9491	0.8705	0.9274
5	0.9295	0.8702	0.9122
Mean ± SD	0.9523±0.0326	0.8708±0.0060	0.9236±0.0085

**Table 10:** Robustness detection.

Perturbation Type	*P*	Change Rate	*R*	Change Rate	mAP@0.5	Change Rate
baseline	0.9330	-	0.7970	-	0.9030	-
motion blur	0.9101	−2.46%	0.8146	+2.21%	0.8940	−0.99%
brightness_contrast	0.9165	−1.77%	0.7979	+0.11%	0.8982	−0.53%
jpeg_compression	0.9240	−0.96%	0.8108	+1.73%	0.9024	−0.07%
rotate_shift_scale	0.9242	−0.95%	0.7968	−0.03%	0.8919	−1.23%
hue_saturation	0.9329	−0.01%	0.8013	+0.54%	0.9013	−0.19%

**Table 11:** Performance comparison of different detection models in defect detection of ampoule bottles.

Category	*P*	*R*	mAP@0.5	mAP@0.5:0.95	*FPS*
YOLOv5 [44]	0.904	0.801	0.866	0.581	227.27
YOLOv7 [45]	0.867	0.462	0.635	0.406	50.76
YOLOv11 [36]	0.935	0.819	0.905	0.622	222
YOLOv8 [36]	0.933	0.797	0.903	0.639	233

**Table 12:** Semi-quantitative comparison results.

Indicator	Integrated Deployment	Separate Deployment
the number of cameras	1	2
light sources and imaging channels	Shared channels	Need to switch channels
image acquisition time	44.1 ms	88.2 ms
system synchronization complexity	Medium	High complexity
calibration workload	Requires only one calibration	Requires two calibrations

## Data Availability

The raw data supporting the conclusions of this article will be made available by the authors upon request.
